# Authors’ Response to Peer Reviews of “Medical Expectations of Physicians on AI Solutions in Daily Practice: Cross-Sectional Survey Study”

**DOI:** 10.2196/56441

**Published:** 2024-03-25

**Authors:** Mara Giavina-Bianchi, Edson Amaro Jr, Birajara Soares Machado

**Affiliations:** 1 Big Data Department, Hospital Israelita Albert Einstein, Sao Paulo, Brazil

**Keywords:** artificial intelligence, adoption, acceptance, opinion, perceptions, survey, expectations, physician, medical survey, qualitative study

*This is the authors’ response to peer-review reports for “Medical Expectations of Physicians on AI Solutions in Daily Practice: Cross-Sectional Survey Study.”*

## Round 1 Review

Dear Reviewers,

We deeply appreciate your time and effort to analyze our manuscript and make suggestions to improve it. We believe it was very helpful and practically all of them were incorporated in this new version. Thank you very much!

### Reviewer AE [1]

#### General Comments

*The manuscript [2] delves into the perspectives of Brazilian physicians on the integration of artificial intelligence (AI) in medical practices through an online cross-sectional survey.*

#### Specific Comments

##### Major Comments

*1. The study purports to evaluate the acceptance of AI by physicians, but the specific types of AI technologies explored remain ambiguous. Are they examining generative AI, natural language processing tools, classical machine learning or other uses of AI?*

Response: There was not a specific technology evaluated, but, most of the times, we were exploring the idea of having a computer-aided solution, such as an AI algorithm, to aid physicians in clinical practice to diagnose, manage, or interpret exams. We introduced this sentence to clarify it in “Methods”: “No specific technology was evaluated. Most of the questions asked about the use of AI algorithms for diagnoses or management of diseases, aiming to address the possible expectations of our target population composed by physicians in clinical practice.”

*2. The phrase “Although scarcely used in real practice” comes across as too assertive. Consider a softer phrasing.*

Response: We modified it to “Although not so frequently used in daily practice yet.”

*3. The methods section should provide a more comprehensive breakdown of the questionnaire’s design process. Which question types were chosen (Likert scale, yes or no, or numerical), and for what reasons?*

Response: Thank you for this suggestion! We modified the “Methods” section to better explain the questionnaire, such as:

“The questionnaire was divided into five sections. The first one was the Informed Consent Term (question 1). Section 2 (questions 2-12) was designed to profile the physicians (sex, age, highest level of education, medical specialty, years since graduation, private versus public sector work, city and state of work, self-assessment knowledge of AI in general, and use of AI solutions in general for daily tasks. Section 3 (questions 13-18) was thought to explore the physicians’ thoughts about AI solutions for diagnosis, management, subsidiary exams interpretation of diseases, as COVID-19, for example, and about the use of AI solutions for diagnosis or treatment of diseases by nurses, physiotherapists or directly by the patient. We also proposed a hypothetical exercise to evaluate physicians’ anxiety feelings and actions taken if him/herself had received a suspicious diagnose of melanoma for one of his or her skin lesions by an AI algorithm. Section 4 (questions 19-24) asked about expected benefits and problems, possible frequency of AI adoption, workload, and utility. Section 5 (questions 25-30) are about physicians’ replacement by AI solutions, financial expectations, possible scenarios of AI and physicians’ disagreements, legal and regulatory aspects. Along with the questions, there were many opportunities for physicians to make comments in an open box about the answers. Physicians could skip to answer any question, thus number of responders could vary along the questionnaire.”

Also, the supplementary file, with the complete questionnaire, was uploaded.

*4. When presenting results, always give raw data (numerator/denominator) along with percentages, especially after statements such as “Most of them described their AI knowledge as intermediate.”*

Response: Thank you, we modified all results and the abstract to give raw data and their percentages.

*5. There is a noticeable omission of a power analysis. How can we ascertain that the sample size sufficiently represents the broader population? The description of the target population needs elaboration.*

Response: As we performed a survey, obviously, there were many questions to be answered by the physicians, but we picked question 21 as the most significant to compare between groups with ≤20 years and >20 years since graduation and to estimate our power level analysis. Question 21 asked how frequent the physicians would adopt AI solutions in their daily practice if they were proven to be reliable and took up to 2 minutes of their time. The possible answers were never, rarely, and sometimes (which we grouped as not favorable) and frequently, most of the times, and always (grouped as a favorable opinion). We estimated that we would have around 35% of favorable responses in the group with >20 years since graduation and 65% in the group with ≤20 years since graduation. Considering an α error of .05 and a β error of .20, we calculated that 48 participants in each group would give us an 80% power level. As we had 163 participants, 103 with >20 years and 60 with ≤20 years, we reached the proposed power level.

However, one other reviewer suggested to divide the years since graduation in 3 groups: ≤10 years, 11-20 years, and >20 years, and repeat the statistical analysis. We did it, and we found that there is a significant difference among the 3 groups (*P*=.0402), as we can see in Table 3.

Our target population was made of physicians who were part of the clinical staff of the Hospital Israelita Albert Einstein and is not intended to represent the entire population of Brazilian physicians. The intention was to point out that the research was done with Brazilian physicians and not to state that the physicians from our hospital represent the Brazilian physician population, because they do not. The aim was to highlight the fact that this was a study conducted in Brazil, which is a geographic region of the world not yet studied in the subject. To clarify that, we changed some of the words, such as from “Brazilian physicians” to “physicians from Brazil,” in the “Introduction.” We included this sentence in “Methods”: “Although physicians of HIAE do not represent the entire population of physicians in Brazil, their answers can give some insights of the subject, since, at this moment, we have none,” and added this sentence in the “Discussion”: “Our target population does not intend to represent the entire population of Brazilian physicians. Even so, a survey performed in one single, large, private hospital can be a way of drawing attention and start a debate about this new subject in Medicine among our physicians, besides capturing their expectations on the topic.”

Brazil is a very large and heterogeneous country, including its health system, and 85% of its 210 million inhabitants rely on public health, which has many more issues than private ones.

##### Minor Comments

*1. The statement “Artificial intelligence (AI) applied to Medicine has been a trending subject in recent years” is preferable over mentioning it as the “hottest topic.”*

Response: We modified it to “Artificial intelligence (AI) applied to Medicine has been a trending subject for the past years.”

*2. In Table 1, the age bracket should read “50-65” as the “50” seems to be missing. Several P values appear without context, for instance: “10. General AI Knowledge (n=164); P=.2565,” “11. Regularity of AI tool usage in daily life (n=164); P=.9792,” and “12. Familiar with medical AI solutions? (n=164); P=.2774.”*

Response: Thank you for pointing out our mistake. All tables were modified as a suggestion of the other reviewer, and a new table was incorporated into the manuscript. The significant *P* values were explained in the footnotes. *P* values that are not significant were removed from the tables.

In “Methods,” we try to clarify our tests, such as the following: “There were two different questions involving statistical analysis. In the first one: ‘does the time since medical graduation matter?,’ the subjects were divided into 3 groups ≤10 years, 11-20 years, or >20 years, according to the answer to question 7 of the questionnaire. In the second, ‘does the sex matter?,’ physicians were divided in male or female, following their answers in question number 3. Statistical analyses between for both analyses were performed using the *χ*^2^ test in Prism software version 6 (GraphPad Software, Inc., San Diego, CA, USA). *P* value <.05 was considered significant.”

Also, the supplementary file, with the complete questionnaire, was uploaded, which can give a better context of the questions.

Tables were also modified to try to improve the questions’ understanding.

*3. Tables 2 and 3 also contain P values that require explanations or clarifications.*

Response: See above.

### Reviewer AJ [3]

#### General Comments

*This paper reports the results of a survey on medical expectations on artificial intelligence solutions in daily practice. The authors argue that it is important to know the opinion that physicians would have as users of these solutions, and the reviewer could not agree more. Therefore, the results of this work may be of interest to the community.*

#### Specific Comments

##### Major Comments

*1. The authors say that these results represent the opinion of Brazilian physicians. Perhaps that is a bit presumptuous, at least without somehow justifying the size of the hospital relative to the Brazilian population. What percentage of the Brazilian population attends this hospital? What percentage of Brazilian physicians works there?*

Response: The intention was to point out that the research was done with Brazilian physicians and not to state that the physicians from our hospital represent the Brazilian physician population, because they do not. The aim was to highlight the fact that this was a study conducted in Brazil, which is a geographic region of the world not yet studied in the subject. To clarify that, we changed some of the words, such as from “Brazilian physicians” to “physicians from Brazil,” in the “Introduction,” and we included this sentence in “Methods”: “Although physicians of HIAE do not represent the entire population of physicians in Brazil, their answers can give some insights of the subject, since, at this moment, we have none.”

Brazil is a very large and heterogeneous country, including its health system, and 85% of its 210 million inhabitants rely on public health, which has many more issues than private ones.

*2. I have not been able to find the supplementary material anywhere. Therefore, I could not review the complete questionnaire.*

Response: We provided the complete questionnaire as a supplementary file. I believe now you will be able to access it. Sorry for that!

*3. The division into <20 years of practice and >20 years of practice does not seem sufficient to this reviewer, since in <20 years of practice you can still have quite senior physicians. I would add an additional division:<10 years, 10-20 years, and >20 years of practice.*

Response: We appreciated your thoughts! We followed your suggestion and incorporated the results into the tables. We found that there was a significant difference in the frequency of intention to use AI solutions according to this new division of years since graduation. Physicians with ≤10 years since graduation are more prone to use it always or most of the times than those with >20 years since graduation, as you will see.

##### Minor Comments

*4. How are the percentages calculated in Table 1? The percentages of every column should sum up to 100.*

Response: There was a missing line in Table 1. Anyhow, the percentages were always calculated within the given column.

*5. Could the authors comment on, if the physicians reported it in the questionnaire, which AI solutions they used in their daily life? Are they used in their personal life or in their work?*

Response: No, the specific app (which uses AI algorithms) in their daily lives was not asked, but we believe it is the same as most of the people in Brazil: Instagram, WhatsApp, Waze, Google Apps, Alexa, Siri, Twitter, and banks app.

We did not ask about algorithms already used in medical practice, because we knew that they were only available for a small part of the target population, such as radiologists, in our hospital.

*6. I assume there is an issue with the color legend for “Work facilitation” in Figure 2.*

Response: Indeed! Thank you again for pointing that out! It was corrected.

*7. I would not only say that physicians think AI will not interfere with the number of appointments. A third of them thinks that AI solutions will increase the number of appointments.*

Response: Agreed! We highlighted that in the results part.

*8. I would include, if possible, a subanalysis of the responses per gender and discuss if there is any differences.*

Response: Very nice suggestion! It was done. We found one statistical difference regarding the acceptance of AI use by other hospital staff (nurses and physiotherapists). Female physicians were more favorable to it than male physicians (*P*=.0079).

Thank you both again!

Best regards.

## Round 2 Review

Dear Reviewer AJ and Editor,

Thank you again for your answer and suggestions to improve our manuscript.

We hope we can address those comments properly.

If there is any other comments or suggestions, please, let us know.

Many thanks again and best regards.

### Reviewer AJ

#### General Comments

*This reviewer thanks the authors for the work done to improve the quality of the paper with this revision. However, I still have some comments.*

#### Specific Comments

##### Major Comments

*1. In the previous review round, I asked about the AI solutions the health care workers used in their daily life.*

Response: We did not ask about the use of specific AI solutions for health care workers in our questionnaire, because we only have a few options, and they are very limited in range and not widespread among specialties. Therefore, we thought only few physicians had already had any contact with AI solutions in our hospital. It is not our reality yet. So, all questions were asked as a hypothetical. Due to this reality, we asked question 12:

“12) Are you aware of any ARTIFICIAL INTELLIGENCE algorithms that have been approved for medical use?

Yes

No

I am not sure”

Anyway, in my opinion, if we would redo the research nowadays, I think that question would be very interesting to pose.

*The authors replied by saying “the specific app (which uses AI algorithms) in their daily lives was not asked, but we believe it is the same as most of the people in Brazil: Instagram, WhatsApp, Waze, Google Apps, Alexa, Siri, Twitter and banks app.”*

Response: This comment was made because we understood that you wanted to know which apps were asked for in question 11, which was not related to medicine or health care:

“11) If there is an option to use ARTIFICIAL INTELLIGENCE for some task in your day-to-day life, how often would you choose to use them?

Never

Rarely

Sometimes

Often

Always

I do not know”

*This reviewer thinks this should be commented somewhere in the manuscript. From this questionnaire question, it seemed that workers have access to true AI solutions in their daily lives. However, these apps the authors mentioned as “AI solutions” use AI in their workflow but are not entirely based on AI and should not be considered “AI solutions.” Without commenting on this, the reader may think that the experience of this population in the use of AI is greater than it really is.*

Response: Thank you. We agree with you. We modified the paragraph in “Methods” that explains Section 2 of the questionnaire to the following:

“Section 2 (questions 2-12) was designed to profile the physicians (sex, age, highest level of education, medical specialty, years since graduation, private versus public sector work, city and state of work, self-assessment knowledge of AI in general (not specific for health care AI solutions), and use of computer or smartphone applications that use AI solutions for daily tasks, such as WhatsApp, Instagram, Facebook, Waze, Google Map, Bank’s app, among others. We did not ask specifically about the use of IA solutions for healthcare in their daily work, only if they were aware of AI solutions in Medicine.”

We did not ask specifically about the use of AI solutions for health care in their daily work, for the reasons explained above.

We also modified a sentence in “Descriptive Results” to mark the difference between AI solutions in general and AI solutions for health care workers:

“Most of the participants use smartphones or computers applications that incorporate AI algorithms for daily tasks outside of work (119/164, 73%) and claim to be aware of AI algorithms applied specific to Medicine (86/164, 52%).”

We also included a sentence in the “Discussion”: “Even so, a survey performed in one single, large, private hospital can be a way of drawing attention and start a debate about this new subject in Medicine among our physicians, besides capturing their expectations on the topic, as AI solutions for healthcare workers are only few, very limited in range and not widespread among specialties in our reality.”

##### Minor Comments

*2. I have not yet been able to access the supplementary material, and the color legend in Figure 2 is still not fixed.*

Response: I will contact JMIRx support to question why you cannot access our supplementary material, once our new Figure 2 is uploaded in the system.

Figure 2 was fixed last time. We uploaded a new version with only 2 colors as we only had 2 answers for “work facilitation”: “yes” and “not alters.” There was no “no” answers, so we did not include “no” in the figure. I hope you can also access both, since we find them necessary for your assessment.

*3. In the text, it appears as P=.079, which is not significant. Please check.*

Response: Thanks again. We corrected the *P* value to .0079 in the text, as it appears in the Table 2 footnote.

*4. The P=.0513 in Table 2 is not significant.*

Response: Yes, we deleted this *P* value in Table 2.

*5. There should be a “Total” column in Table 1.*

Response: We inserted a “TOTAL column n/(%)” as asked. Thank you.

## Abbreviations

AI: artificial intelligence
